# New frontiers in endovascular therapies for locally advanced hepatocellular carcinoma

**DOI:** 10.1590/0100-3984.2020.0027

**Published:** 2021

**Authors:** Riccardo Inchingolo, Stavros Spiliopoulos, Alessandro Posa, Tiago Kojun Tibana, Thiago Franchi Nunes, Riccardo Memeo

**Affiliations:** 1 Interventional Radiology Unit, "F. Miulli" Regional General Hospital, Acquaviva delle Fonti (BA), Italy.; 2 Radiology Department, King's College Hospital, London, UK.; 3 2nd Radiology Department, National and Kapodistrian University of Athens, Athens, Greece.; 4 Department of Radiology, Fondazione Policlinico Universitario A. Gemelli, Rome, Italy.; 5 Department of Interventional and Vascular Radiology, Universidade Federal de Mato Grosso do Sul (UFMS), Campo Grande, MS, Brazil.; 6 Unit of Hepato-Pancreatic-Biliary Surgery, "F. Miulli" Regional General Hospital, Acquaviva delle Fonti (BA), Italy.

**Keywords:** Carcinoma, hepatocellular/therapy, Fibrosis, Liver/pathology, Medical oncology/methods, Chemoembolization, therapeutic, Liver neoplasms/radiotherapy, Carcinoma hepatocelular/terapia, Fibrose, Fígado/patologia, Oncologia/métodos, Quimioembolização terapêutica, Neoplasias hepáticas/radioterapia

## Abstract

Hepatocellular carcinoma is the most common primary malignant liver tumour and is a leading cause of death worldwide. Despite the advent of screening programmes, most cases of hepatocellular carcinoma are diagnosed late (in an advanced stage) which precludes curative treatments such as surgery and ablation. Therefore, intra-arterial locoregional treatments now play a central role in the management of advanced hepatocellular carcinoma, such treatments ranging from trans-arterial chemo-embolisation to the more recently developed trans-arterial radio-embolisation technique. In this essay, we discuss the state of the art of intra-arterial treatment for locally advanced hepatocellular carcinoma and the future directions for such treatment.

## Abstract (pt)

O carcinoma hepatocelular é o tumor hepático maligno primário mais frequentemente observado, sendo uma das principais causas de mortalidade mundial. Apesar do advento dos programas de triagem, na maioria dos casos o diagnóstico tardio está associado a um estágio avançado da doença que impede o tratamento curativo, como cirurgia ou ablação. Assim, os tratamentos locorregionais intra-arteriais têm agora um papel central no gerenciamento avançado do carcinoma hepatocelular, passando por quimioembolização transarterial e radioembolização. Neste ensaio, discutimos o estado da arte do tratamento intra-arterial atualmente disponível para o carcinoma hepatocelular localmente avançado e suas direções futuras.

## INTRODUCTION

Hepatocellular carcinoma (HCC) is the most common primary liver cancer, accounting for 75% to 90% of all primary liver tumours, depending on the country, and is still a leading cause of death worldwide^(1)^. A common finding in patients with liver cirrhosis is multi-nodular HCC, which is classified as an intermediate-stage (stage B) tumour in the Barcelona Clinic Liver Cancer (BCLC) staging system^(2)^. Multi-nodular HCC that is deemed neither resectable nor amenable to curative ablative treatments, due to portal vein thrombosis-chemical or neoplastic-albeit with preserved liver function, can be defined as locally advanced, a definition that encompasses the intermediate (BCLC-B) stage and the advanced (BCLC-C) stage. Because patients with multi-nodular HCC have a poor prognosis, they can be submitted to locoregional treatment with endovascular techniques, in order to extend overall survival and increase overall disease control rates.

## TRANS-ARTERIAL CHEMO-EMBOLISATION

Trans-arterial chemo-embolisation (TACE) is the gold-standard treatment for intermediate-stage HCC^(2)^. The effectiveness of TACE is attributable to the combination of the cytotoxic effect of the chemotherapeutic agent and the ischaemic effect of the embolic micro-spheres, which are delivered only to the target lesions^(3)^.

Various chemotherapeutic drugs have been studied and used in TACE treatments, doxorubicin being the one most widely used, and studies have shown that the combined use of more than one chemotherapeutic drug is more efficacious than is monotherapy^(4)^. Although it is recommended that TACE treatments be repeated at regular intervals, there is no consensus on or guidelines regarding the appropriate interval between TACE sessions or the appropriate number of sessions, the choices of which are therefore left to the interventional radiologist. However, it is commonly considered advisable to discontinue TACE if the HCC shows no response or if there is disease progression after two or three TACE sessions^(5)^. In addition, TACE is generally considered a palliative treatment, a complete response being achieved in only 30-40% of cases, with high rates of local recurrence, mostly due to microsatellitosis^(5)^.

The embolising micro-spheres used in TACE can be of various sizes. Although large-calibre micro-spheres (> 300 µm) can create ischaemia, their use may be associated with premature embolisation of the feeding artery (i.e., before the chemotherapeutic agent can be completely effective). Premature arterial embolisation may damage the blood vessel, prevent subsequent tumour-directed treatment, and cause hypoxia-induced neoangiogenesis. In an animal model of liver cancer, infusion of micro-spheres with a calibre of 100-300 µm resulted in their delivery into the tumour or near its margins, justifying their use for precise drug delivery or embolisation^(6)^. Although many institutions recommend using such micro-spheres in the TACE procedure, the choice of micro-sphere calibre depends on many factors, including tumour size, feeding artery diameter, personal preference/experience, and the presence or absence of an arteriovenous shunt, which increases the risk of pulmonary complications^(6,7)^.

**Figure 1:**
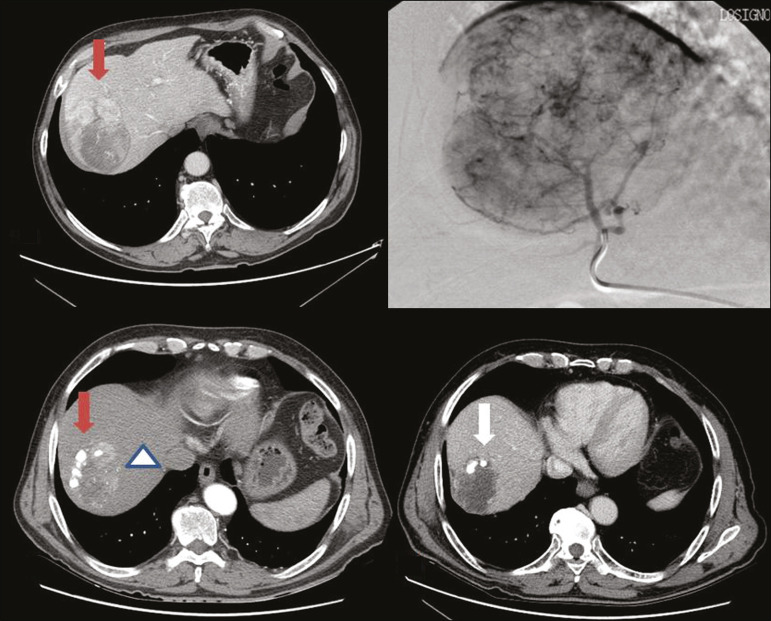
Conventional TACE of a large HCC. Contrast-enhanced computed tomography scan of a 71-year-old patient with liver cirrhosis and a large (8 cm) HCC involving the right liver lobe (red arrow). Right hepatic artery branch digital subtraction angiography showing the hypervascular lesion. Follow-up contrast-enhanced computed tomography scan, acquired one month later, demonstrating a partial response, with tumour necrosis (white arrow), lipiodol deposition, and residual tumour in the lower part of the nodule (arrowhead).

One of the greatest points of debate related to the use of TACE is regarding the approach: on one side, there is conventional TACE, which involves the infusion of an emulsion of the chemotherapeutic drug and iodised oil, which acts as a drug carrier, has a good visibility under fluoroscopy, and is taken up by HCC cells, followed by the introduction of inert embolising micro-spheres (Figure 1); on the other side, there is drug-eluting bead TACE (DEB-TACE), in which the chemotherapeutic drug is preloaded into the embolising micro-spheres (via a chemical link with sulphonate groups) and is then slowly released into the HCC blood supply in a sustained manner, prolonging administration of the drug and avoiding a peak in its plasma concentration^(2-4)^. Various studies have compared those two approaches, most finding that neither was superior to the other. However, one randomised trial showed that the use of DEB-TACE was associated with higher rates of an objective complete response and overall disease control, as well as with a lower rate of adverse effects, although the differences were not statistically significant^(5)^.

**Figure 2:**
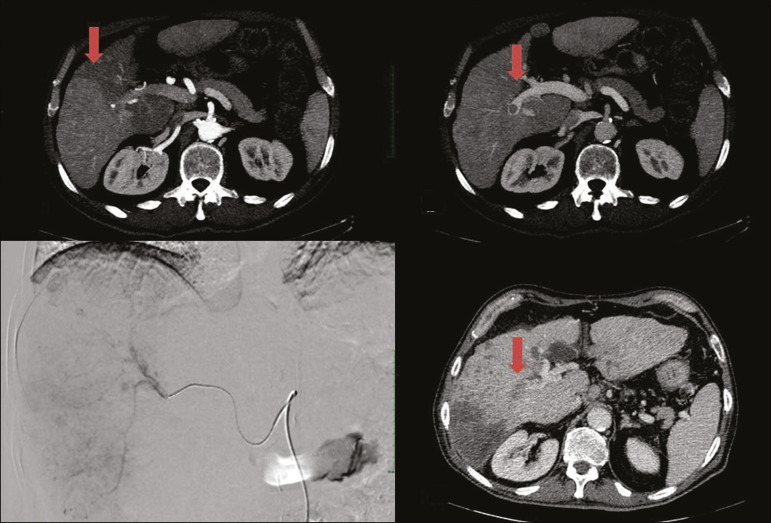
DSM-TACE of multi-nodular HCC. Contrast-enhanced computed tomography scan of a 75-year-old patient with liver cirrhosis and a multi-nodular HCC involving the right liver lobe, showing enhancement in the arterial phase (arrow). Note the right portal vein thrombosis (arrow). Right hepatic artery digital subtraction angiography showing the multi-nodular hypervascular lesion. Follow-up contrast-enhanced computed tomography scan, acquired one month later, demonstrating a partial response, with tumour necrosis and a thrombus remaining in the right branch of the portal vein (arrow).

**Figure 3:**
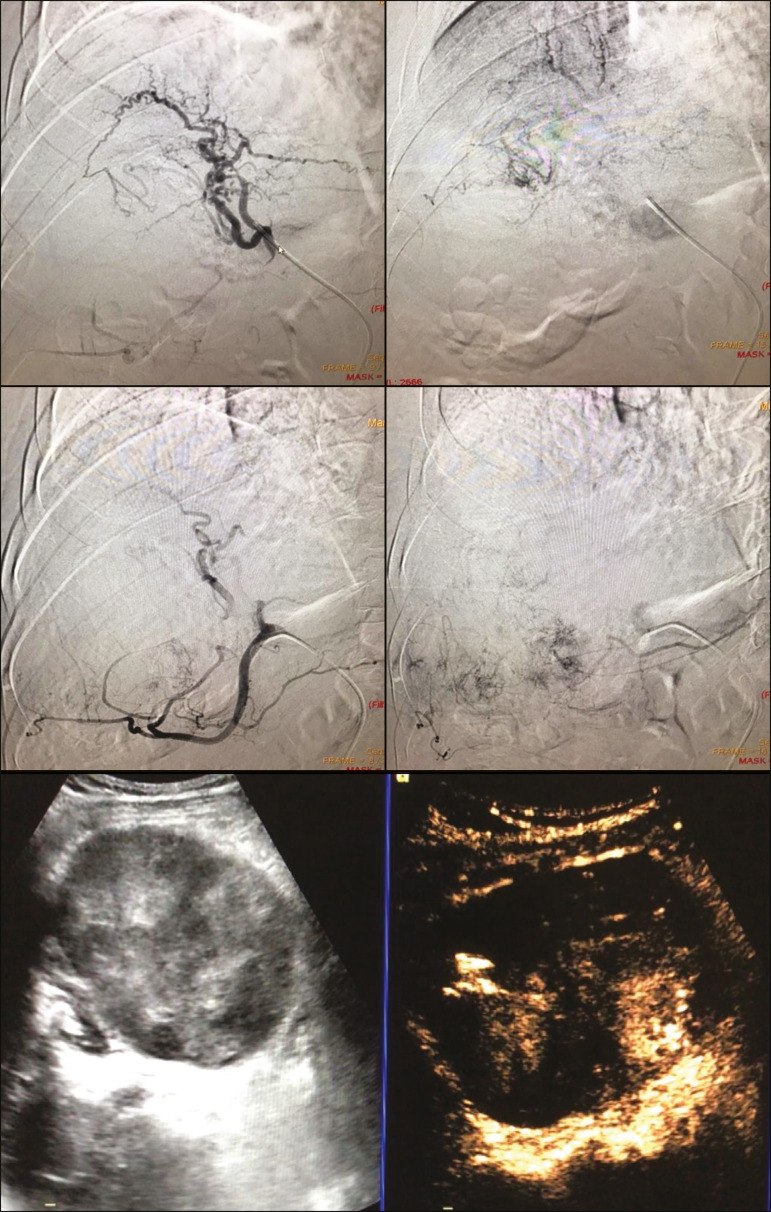
A 54-year-old male patient with alcoholic cirrhosis presenting with an HCC measuring 10 cm and located in the inferior left medial segment (segment IVb) of the liver. DEB-TACE was performed with HepaSphere 150-200 µm (Merit Medical Systems, South Jordan, UT, USA) loaded with doxorubicin 75 mg. Arteriography demonstrating the tumour blood supply originating from branches of the right hepatic artery and lateral branches of the gastro-duodenal artery. Microbubble contrast-enhanced ultrasound performed 24 h after the procedure, showing necrosis in > 90% of the embolised tumour.

**Figure 4:**
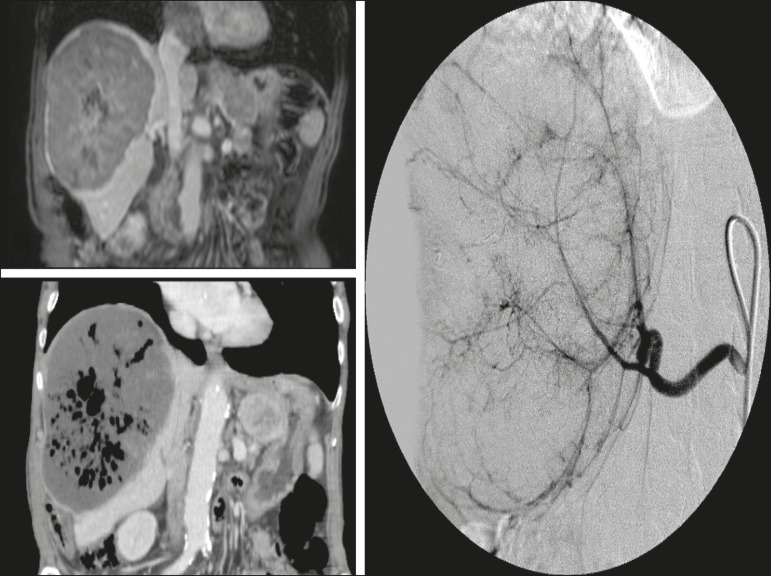
An 82-year-old male patient with an HCC, of the fibrolamellar subtype, measuring 18 cm and located in the right postero-inferior and postero-superior lateral segments (segments VI and VII, respectively) of the liver, as seen on an unenhanced magnetic resonance imaging scan. DEB-TACE of the liver tumour was performed with two vials of HepaSphere 150-200 µm (Merit Medical Systems, South Jordan, UT, USA) loaded with doxorubicin 75 mg. The patient evolved to an excellent general health status, with no signs of infection. A follow-up computed tomography scan, acquired one month later, showing 100% necrosis with intratumoural gas related to the tumour necrosis.

The indications for TACE have been expanded. The introduction of degradable starch micro-sphere TACE (DSM-TACE) has made it possible to use TACE to treat advanced-stage (BCLC-C) HCC as well as intermediate-stage HCC that progresses after the use of locoregional therapies. According to various guidelines^(3-5)^, such HCCs should be treated with the multi-kinase inhibitor sorafenib. However, sorafenib is associated with major adverse effects and its use is therefore contraindicated in or considered unacceptable by some patients. Such patients could be submitted to DSM-TACE, which uses starch micro-spheres that are degraded rapidly (in 25-40 min) by hepatic alpha-amylases. In DSM-TACE, there is only temporary occlusion of the arterial hepatic blood flow, decreasing the ischaemic embolic effect on the liver parenchyma and minimising the immediate wash-out of the chemotherapeutic drug, thereby allowing superselective administration and reducing the occurrence of systemic side effects (Figures 2, 3 and 4).

## TRANS-ARTERIAL RADIO-EMBOLISATION

Another treatment for locally advanced HCC is trans-arterial radio-embolisation (TARE), also known as selective internal radiation therapy. Although it has yet to be included in the BCLC guidelines, TARE is most commonly used in intermediate-stage (BCLC-B) or advanced-stage (BCLC-C) HCC that is ineligible for treatment with TACE or sorafenib^(7)^. The mechanism of action of TARE is based on the local action of micro-spheres loaded with yttrium-90, which emits beta radiation when decaying to zirconium-90. Beta emission is in the range of few millimetres, which limits exposure of healthy liver parenchyma to radiation, thus lowering the risk of radiation-induced liver failure^(7)^. Pre-procedural planning is mandatory in cases in which yttrium-90 TARE is used: it requires a dedicated angiographic procedure in which there is intra-arterial administration of macro-aggregates of human serum albumin labelled with technetium-99m in the most ideal position, immediately followed by a single-photon emission computed tomography examination to assess extra-hepatic distribution of the micro-spheres and to quantify the hepato-pulmonary shunt fraction; and non-target vessels should be coil-embolised selectively during the pre-procedural planning, to prevent extra-hepatic passage of yttrium-90 during the treatment session. Yttrium-90 TARE can be performed with glass or resin permanent micro-spheres. In recent years, other types of isotopes have been employed in TARE, including iodine-131 lipiodol, which avoids the embolic effect of micro-spheres that could impair liver function in advanced-stage HCC, and holmium-166, which is loaded into micro-spheres made of poly-L-lactic acid and also emits gamma radiation, allowing scintigraphy evaluation of its distribution. Holmium-166 is also highly paramagnetic and is therefore visualisable during magnetic resonance imaging^(3,7)^. To our knowledge, there have been no studies comparing these various isotopes and micro-spheres. Therefore, further studies are needed.

## COMBINED TREATMENTS

Another option in patients with HCC is combined treatment with thermal ablation and TACE, combining the thermal ablative effect of radio waves or microwaves with the ischaemic and cytotoxic effects of TACE, dramatically increasing the therapeutic effect. In fact, this approach is typically recommended for single, large (> 3 cm) unresectable early- or intermediate-stage HCCs, with a great efficacy in lesions < 5 cm, with better results in terms of disease-free survival and complete response rates when compared to radiofrequency ablation or TACE alone. However, this combined treatment has yet to be standardised, and there is still no consensus as to whether the thermal ablation should be performed before or after TACE, as well as whether both treatments should be performed in a single-step fashion or in a sequential manner (with an interval between the two from 24 h to several days) to achieve the optimal synergistic effect^(8)^. The other advantage of combined treatment is its usefulness in treating lesions in unfavourable locations, such as peri-cholecystic and sub-capsular lesions, as well as hypovascular lesions due to the hyperaemic effect of thermal ablation, although it is also of great help in treating post-thermal ablation complications, such as bleeding (especially in patients with low platelet counts), with the same TACE micro-spheres^(9,10)^.

## CONCLUSION

In summary, future research should focus on the combination of locoregional therapies. High-quality data from multi-centre randomised controlled trials are needed in order to investigate the possibility of improving overall survival in unresectable HCC, as well as to draw comparisons among trans-arterial embolisation, DEB-TACE, and conventional TACE. Because TARE is a very promising therapy, the initial failure to improve survival in patients with intermediate- or advanced-stage HCC should not discourage investigators: well-designed trials with better patient selection would certainly help define its role in HCC treatment.
